# Supplementary material for: Circ-ERC2 Is Involved in Melatonin Synthesis by Regulating the miR-125a-5p/MAT2A Axis
Source: Int J Mol Sci. 2022 Dec 7;23(24):15477. doi: 10.3390/ijms232415477 (PMC9778726; doi:10.3390/ijms232415477)
Supplement: Supplementary file 1 [file ijms-23-15477-s001.zip › ijms-2043966 supplementary Figure.pdf]

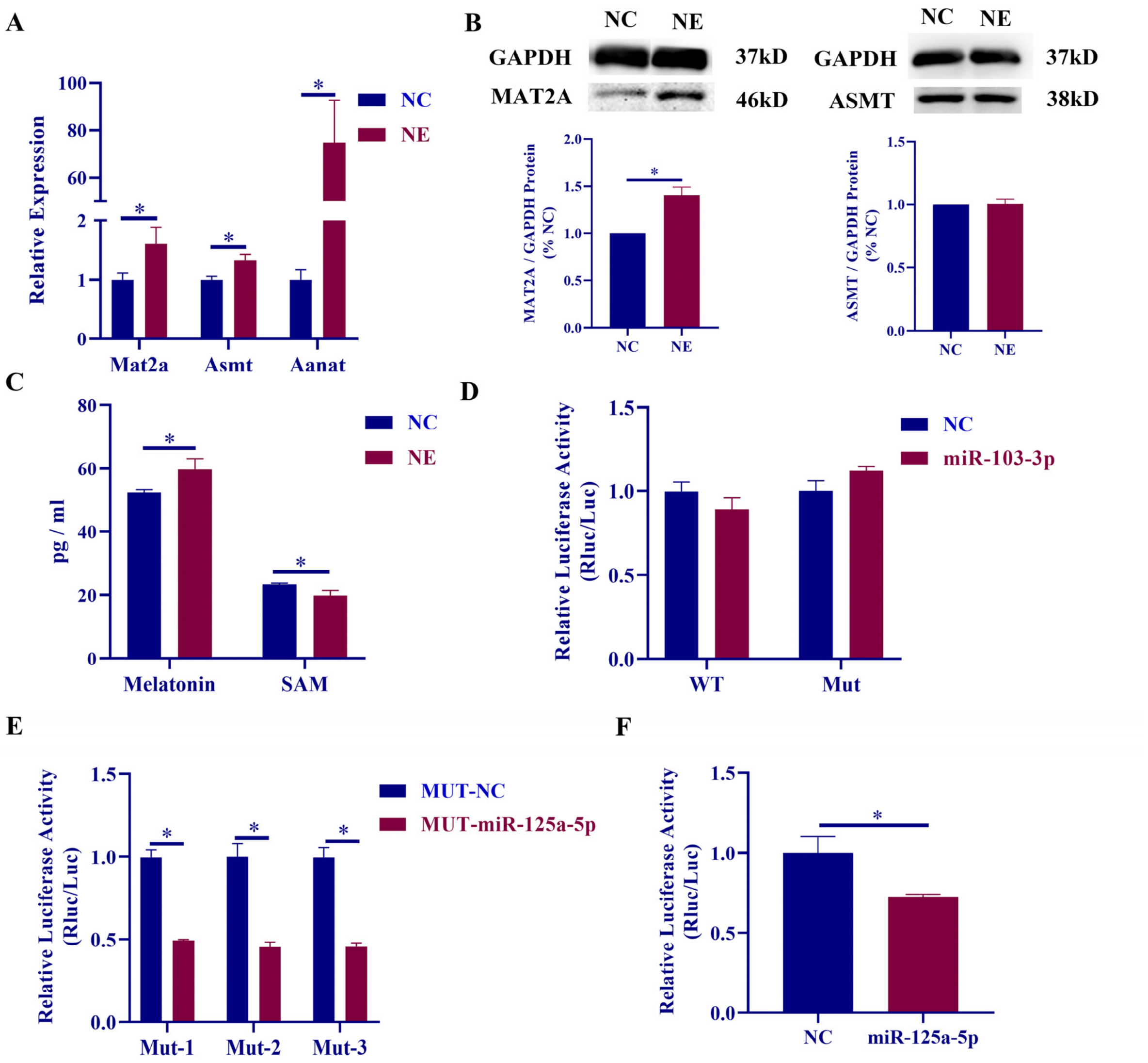

Figure S1. Experimental supplement results. (A) Relative expression of mat2a, asmt and aanat in pineal cells of rats before and after NE treatment. (B) Relative expression of MAT2A and ASMT proteins in pineal cells of rats before and after NE treatment. (C) Relative expression of melatonin and S-adenosine methionine (SAM) in the supernatant of rat pineal cells before and after NE treatment. (D) Dual luciferase reporter assay results for miR-103-3p, the wild-typemat2a mRNA 3'-UTR and a mutant mat2a mRNA 3'-UTR. (E) Dual luciferase reporter assay results for miR-125a-5p, the mutant mat2a mRNA 3'-UTR. (F) Dual luciferase reporter assay results for miR-125a-5p, the mutant circ-ERC2. *, $P < 0.05$.
